# Inhaled CpG increases survival and synergizes with checkpoint inhibition in lymphangioleiomyomatosis

**DOI:** 10.1101/2023.02.06.527331

**Published:** 2023-02-07

**Authors:** Mayowa M Amosu, Jacob C McCright, Bennett E Yang, Juan Grano de Oro Fernandez, Kaitlyn A Moore, Havish S Gadde, Michele L Kaluzienski, Katharina Maisel

**Affiliations:** 1 Fischell Department of Bioengineering, University of Maryland, College Park, MD 20742

**Keywords:** TLR agonist, regulatory T cells, metastatic murine LAM, combination immunotherapy

## Abstract

**Rationale:**

Lymphangioleiomyomatosis (LAM) is a devastating disease primarily found in women of reproductive age that leads to cancer-like cystic destruction of the lungs. Recent work has shown that LAM causes immunosuppression and that checkpoint inhibitors can be used as LAM treatment. IN lung cancer, TLR agonist, in particular TLR9 agonist CpG has been shown to be effective.

**Objectives:**

Here we investigate the use of TLR9 agonist CpG as LAM immunotherapy in combination with checkpoint inhibitor, anti-PD1 and assess induced changes in anti-LAM immunity.

**Methods:**

We used a murine model of metastatic LAM to determine survival after intranasal treatment with TLR9 agonist CpG at two doses and in combination the checkpoint inhibitor immunotherapy, anti-PD-1. We used histology and flow cytometry to assess overall inflammation as well as changes in the immune response upon treatment.

**Measurements and Main Results:**

We found that local administration of CpG enhances survival in a murine model of LAM and that a lower dose more effectively balanced the inflammation induced by CpG with the anti-LAM therapeutic benefits. We also found that CpG reduces regulatory T cell infiltration in LAM lungs and that CD4 helper T cells are skewed toward pro-inflammatory phenotypes. We also found that CpG treatment is effective in both early stage and progressive disease and that CpG is synergistic with previously tested anti-PD1 therapy.

**Conclusions:**

We have found that TLR9 agonist CpG can be used as LAM immunotherapy and effectively synergizes with anti-PD1 therapy in LAM.

## INTRODUCTION

Lymphangioleiomyomatosis (LAM) is a progressive rare lung disease, primarily affecting women of child-bearing age (1). LAM patients can experience a range of debilitating symptoms from shortness of breath, lung collapse, and eventual respiratory failure. LAM is characterized by the persistent abnormal growth of smooth muscle-like cells (LAM cells) and the formation of thin-walled cysts predominantly in the lungs, with involvement of the surrounding lymphatics and kidneys. LAM is caused by inactivating mutations in tumor suppressor genes tuberous sclerosis complex 1 and 2 (TSC1 / TSC2) that negatively regulate cell growth through inhibition of a signaling protein complex called mammalian target of rapamycin (mTOR). Currently, sirolimus (rapamycin) is the only FDA approved drug for LAM (2), which inhibits the constitutively activated mTOR complex thus slowing disease progression (3,4). However, rapamycin does not promote disease regression or lung regeneration (5,6). Furthermore, patient responses to sirolimus are incomplete and not always well tolerated for life-long use (5). Therefore, there is an urgent need for true curative treatments that can both eliminate LAM nodules and restore healthy lung function in patients suffering from LAM. LAM is designated as a neoplastic growth, and recent research debate suggests it has similarities to cancer (7). Immunosuppression is a common feature of many cancers, including lung cancers(8)(9), which over time conditions immune cells to ignore the tumor cells thus promoting cancer progression (10–12). In human LAM tissues, a lack of T-cell infiltration suggests immunosuppression due to unresponsiveness of lymphocytes to LAM antigens (13). LAM lungs typically have elevated levels of secreted factors like monocyte chemoattractant protein 1 (MCP-1) (14) which may contribute to recruitment of immunosuppressive cells (15,16). Prior work by us and others has also shown that LAM causes overexpression of checkpoint molecules PD-1 and CTLA-4 on T cells and LAM nodules have elevated expression of PD-1’s binding partner, PD-L1 (17,18). Anti-PD1/PD-L1 and anti-CTLA-4 therapies were shown to enhance survival in murine models of LAM (17,18), suggesting that countering immunosuppression in LAM could be an effective strategy for immunotherapy.

In addition to the previously investigated checkpoint inhibitor treatments, reducing immunosuppression in LAM could be accomplished by reactivating antigen presenting cells (APCs) in the local tumor environment. Recent studies investigating targeted activation of APC’s in cancer show enhanced anti-tumor immunity through improved T cell responses (19), and increased responsiveness of immunosuppressed myeloid cells to immunotherapy (20). Local tumor treatments inducing APC activation have also been shown to stimulate systemic anti-tumor immunity and regression of distant tumors (21). APCs are particularly responsive to molecules that mimic microbial pathogens to stimulate toll-like receptors (TLRs). When engaged through TLR activation, APCs like dendritic cells and macrophages secrete inflammatory cytokines and promote the differentiation of cytotoxic T cells (22,23). Several TLR agonists have received FDA approval for treatment of various cancers. For example, BCG is a TLR4 agonist that is used as immunotherapy for bladder cancer. Currently, there are 5 clinical trials investigating the use of TLR3, and TLR7/8 agonists as cancer vaccine adjuvants (NCT04364230), monotherapy (NCT04819373), or in combination with immunotherapy (NCT04278144, NCT03486301, NCT04840394). Three active clinical trials are also investigating targeted delivery of TLR9 agonists for treatment of various cancers (NCT02668770, NCT05607953, NCT04935229). TLR9 agonists, like CpG, have been approved for use in humans as adjuvants in vaccination and CpG in particular has been explored as therapy in multiple cancers including non-small cell lung cancer (24–26). TLR9 activation has also been shown to be synergistic with immune checkpoint inhibition, making resistant tumors more responsive to therapy (27–31). Given the current efforts at leveraging TLR9 agonist use in cancer therapy, we propose to investigate its potential for therapy in LAM. Here, we investigate the efficacy of TLR9 agonist, CpG with or without simultaneous checkpoint inhibition as immune activating treatments for LAM and assess how CpG modulates anti-LAM immunity.

## METHODS

### Mice

7 to 9-week old female C57BL6/J mice (Jackson Laboratory) were used for all studies. Study protocols were approved by the UMD Institutional Animal Care and Use Committee (IACUC).

### LAM induction

TSC2-null kidney-derived epithelial tumor (TTJ) cells, were acquired from the Krymskaya Lab at the University of Pennsylvania (17). TTJ cells (5.0 × 10^5^) were administered in 100 μL of PBS via tail vein injection to induce LAM disease in 8–9 week old female mice. Lung lesions typically develop within 3–7 days.

### Survival Studies

Mice received CpG class B (5 μg/10 μg) 2x/week (Invivogen, tlrl-1826–5), in 50 μL of PBS, intranasally starting 4 days post LAM induction. Mice receiving combination therapy, received intranasal CpG and 300 μg anti-PD-1 antibody (BioXCell, BE0146-A0) in 200 μL of PBS via intraperitoneal (IP) injection 2x/week. Control groups received 50 μL of PBS intranasally and 300 μg of rat IgG isotype control (BioXCell, BE0089) by IP injection. Mice were monitored for symptoms of disease and body weight changes and removed from studies based on experimental timing or humane endpoints. At endpoint, mice were euthanized and lung tissues collected for histological analysis.

### Histology

Tissues were fixed overnight in 4% PFA at 4°C, embedded in paraffin wax, sectioned at 5 μm, and stained with hematoxylin and eosin (H&E). LAM nodule counts, average nodule diameters, and general tissue inflammation were quantified using QuPath Imaging software.

### Flow Cytometry

**Figure E1:**
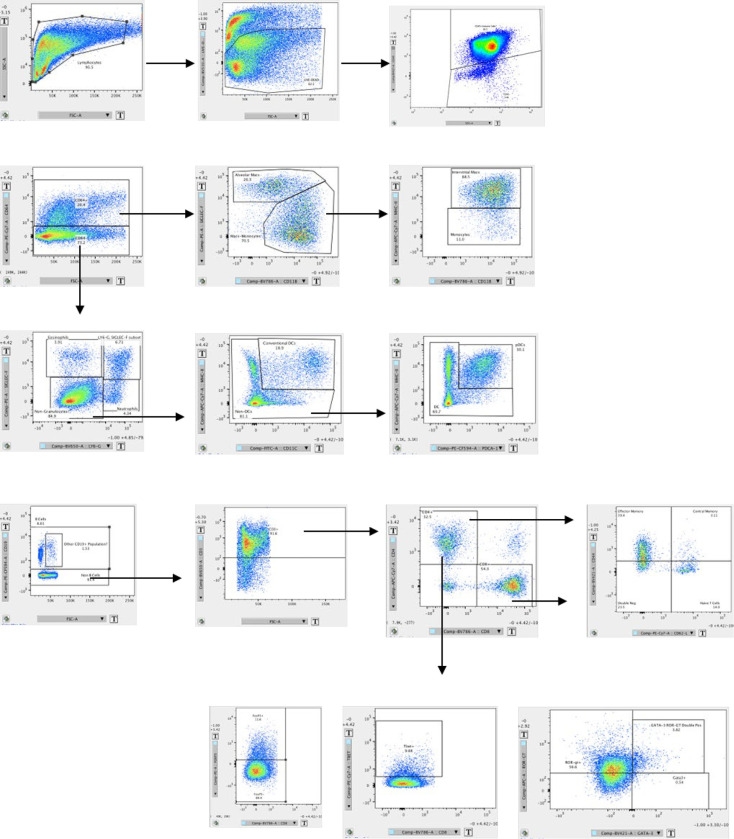
Flow Cytometry Gating Strategy

After LAM induction, mice received a single intranasal treatment dose of CpG (5 μg or 10 μg) in 50 μL PBS or 50 μL of PBS as a vehicle control. At 1 and 3 days post treatment, lung tissues were perfused and collected for flow cytometric analysis. Left lung lobes were dissociated by enzymatic digestion with 1 mg/mL collagenase D (Roche, 11088882001), and 1 mg/mL collagenase IV (Worthington Biochemical, LS004188) in DMEM with 5% FBS for 1 hour. Single cell suspensions were stained to identify eosinophils (CD64−, Siglec F+, Ly6G−), neutrophils (CD64−, Siglec F−, Ly6G+) dendritic cells (CD11c+ MHC-II^high^), alveolar macrophages (CD64+, SiglecF^high^, CD11b−), interstitial macrophages (CD64+, SiglecF^low^, CD11b+, MHC-II+), B Cells (CD19+ or B220+ ), T Cells (CD3+, CD4+ or CD8+), regulatory T Cells (CD3+, CD4+, FoxP3+), Th1 T cells (CD3+, CD4+, FoxP3−, Tbet+), Th2 T cells (CD3+, CD4+, FoxP3−, Gata3hi), and Th17 T Cells (CD3+, CD4+, FoxP3−, ROR-γt+). Cells were analyzed by flow cytometry using a BD FACSCelesta (BD Biosciences) and FlowJo (FlowJo LLC). Gating strategies can be found in the data supplement (Fig E1).

### ELISA

For cytokine analysis, lungs (smallest right lobe) were snap-frozen and stored at −80°C. For tissue homogenates, snap-frozen lungs were homogenized at 4°C at 20% w/v in protein extraction reagent (T-PER, Thermo Fisher Scientific) containing Halt^™^ Protease Inhibitor Cocktail (Thermo Fisher Scientific). Lung tissue homogenates were centrifuged at 15,000 g for 10 min at 4°C. Supernatants were aliquoted and stored at −80°C. Supernatants were diluted 1:10 with 1% BSA ELISA assay diluent and ELISA was performed according to manufacturer’s instruction (ELISA MAX^™^ Standard Set, Biolegend).

### Statistical Analysis

Data were analyzed using Graphpad Prism (GraphPad, La Jolla, CA). Survival studies were assessed using the log-rank (Mantel-Cox) test. Unpaired Welch t tests were used to compare pairs of samples or groups. Mann–Whitney test was used to compare the groups of data that failed to satisfy parametric assumptions. Brown-Forsythe and Welch One-way ANOVA were used for comparisons between ≥3 groups. Differences were considered statistically significant when p < 0.05. *p<0.05, **p<0.01, ***p<0.001, ****p<0.0001.

## RESULTS

### Treatment with Intranasal CpG Increases Median Survival in Murine LAM and is dose dependent

**Figure 1.**
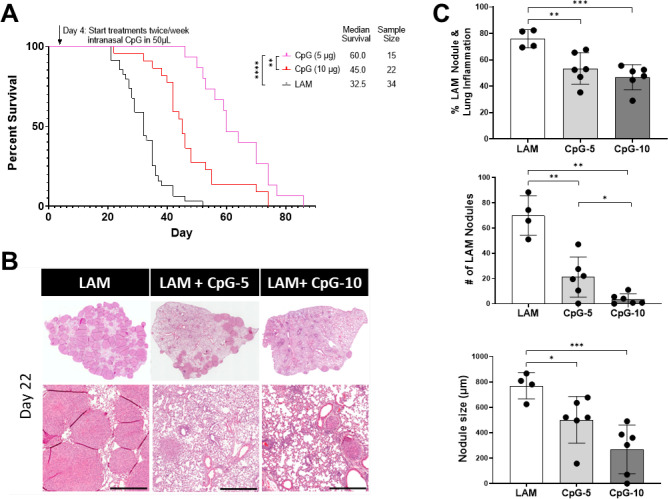
Intranasal CpG increases survival in murine model of metastatic LAM, decreases tissue-wide inflammation and overall LAM nodule burden. **A)** Aggregate data from three survival experiments LAM n=34, CpG (10ug ) n=22, CpG (5ug ) n=15. **B)** Representative H&E stained histological images of mouse lungs from treatment groups: untreated LAM control, CpG 5 μg, and CpG 10 μg. Scale bar = 500 μm. **C)** Quantification of LAM nodules and Inflamed tissue regions between treatment groups 22 days post LAM induction and after 6 total treatment administrations. 3 sections analyzed per mouse. Statistical analysis was performed using log-rank test (survival studies) and one-way ANOVA (histological analysis). * p<0.05, ** p<0.01, *** p<0.001, **** p<0.0001.

To determine the therapeutic benefit of TLR9-agonist CpG in LAM disease, we performed survival studies comparing the effect of administering routine doses of intranasal CpG during the course of LAM disease in our metastatic mouse model. We found that intranasal CpG increases survival in murine LAM from 32.5 to 45 days (Fig 1A) and that this effect is dose dependent, with lower dose CpG (5μg) resulting in higher median survival (60 days) than higher dose CpG (10μg, 45 days). We conducted a cross sectional study after 3 weeks (Day 22) and 6 total treatment administrations to investigate the effect of treatment with CpG in LAM lungs at a point where the treatment groups begin to diverge in survival studies. Histological analysis of these tissues showed that treatment with CpG slows the development of LAM-like nodules in mice as indicated by the reduced tumor burden and nodule sizes in treated vs untreated lungs (Fig 1B). We found that higher dose CpG (10ug), significantly decreased the nodule count, more that the lower dose (5ug), which was surprising given the improvement in overall survival with lower dose CpG (Fig 1C). However, when we considered that at both doses, the combined measure of tumor burden and inflammation are similar (Fig 1C), we found that higher dose CpG causes higher levels of inflammation and may actually contribute to increased morbidity in mice with LAM. Consistent with this assessment, the histological images in the higher dose CpG show considerable immune cell infiltration and thickened alveolar walls compared to lower dose CpG (Fig 1B).

### Intranasal CpG modulates Immune cell infiltration in LAM Lungs

**Figure 2.**
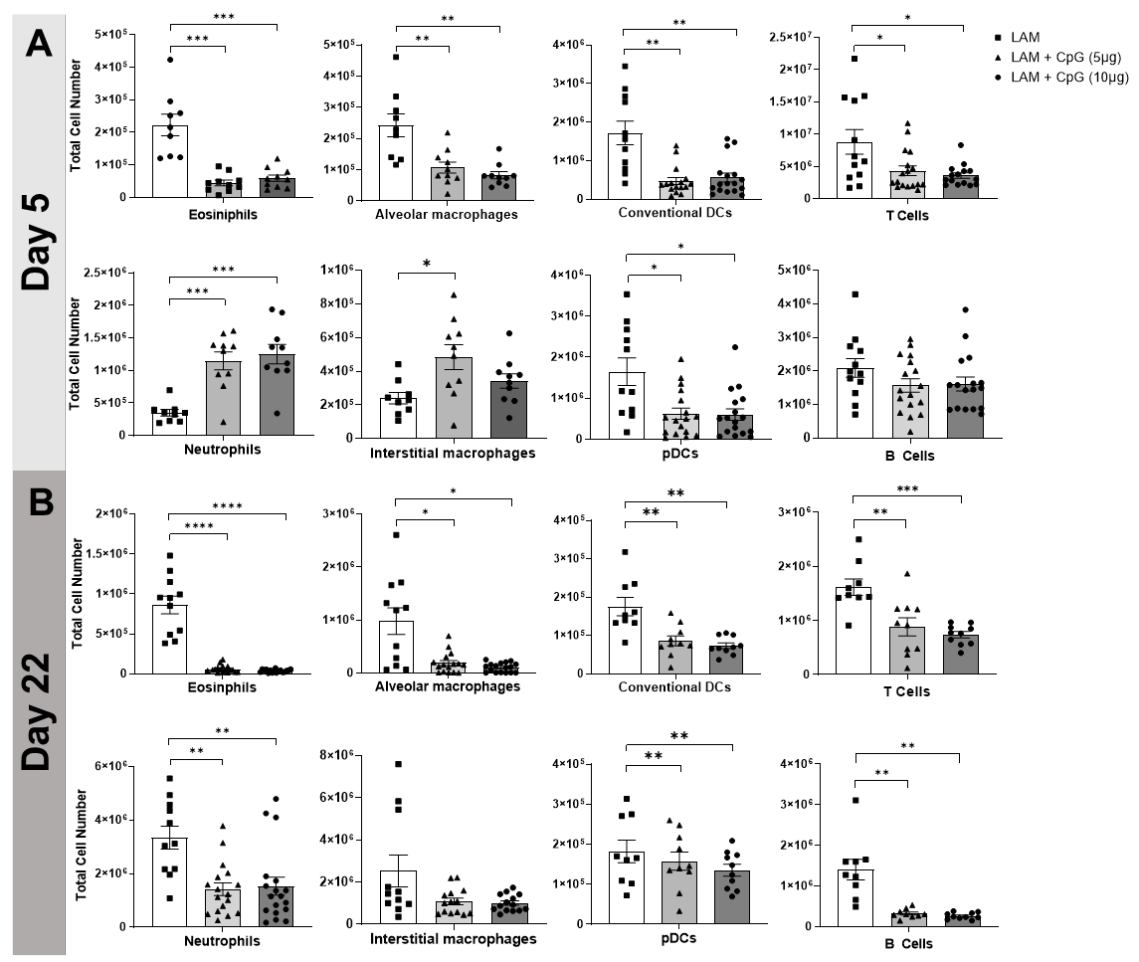
CpG treatment modulates immune cell infiltration in LAM. Data shows total cell numbers in the left lung lobe of granulocytes and antigen presenting cells at day 5 or day 22 after LAM induction and 1 or 18 days respectively after CpG treatment has started. Data is representative of n= 2+ experiments and n>8 mice. Statistical analysis was performed using one-way ANOVA. * p<0.05, ** p<0.01, *** p<0.001, **** p<0.0001.

We next sought to evaluate the effects of CpG treatment on immune cell infiltration in LAM. We conducted flow cytometry analysis of immune cells at two cross-sectional timepoints, day 5 and day 22, of our initial survival study. We found that after the first dose of CpG, given early in the course of disease on day 5, populations of eosinophils, alveolar macrophages, dendritic cells, and B and T cells were significantly decreased at both low and higher dose CpG. In contrast, both neutrophils and interstitial macrophages, and monocytes were increased in response to CpG treatment (Fig 2A, **Supplementary Figure 2**). By day 22, CpG treatment in LAM mice resulted in decreased cell counts in all identified immune populations except B cells and interstitial macrophages when compared to counts in untreated LAM controls (Fig 2B). Comparing the expansion of immune cells within treatment groups between Day 5 and Day 22, we found up to 5 fold increases in T and B cell counts in CpG treated mice compared to up to 10 fold increases in disease controls (Figures E3–4). Between Day 5 and Day 22, untreated mice had greatest expansion of interstitial macrophages and cDCs, while CpG treated mice had greatest expansion in cDCs and T Cells (**Supplementary Figures 3–4**). B cells in untreated mice were the only cell population with little expansion from Day 5 to 22. In CpG treated lungs, there was no expansion in eosinophil, alveolar macrophage and monocyte populations. This suggests that CpG stimulates inflammation in the tissue, and recruits a different assortment of cells while restraining the inflammation seen in untreated LAM.

### Intranasal CpG shifts T Cells toward inflammatory phenotypes and reduces regulatory T cells

**Figure 3.**
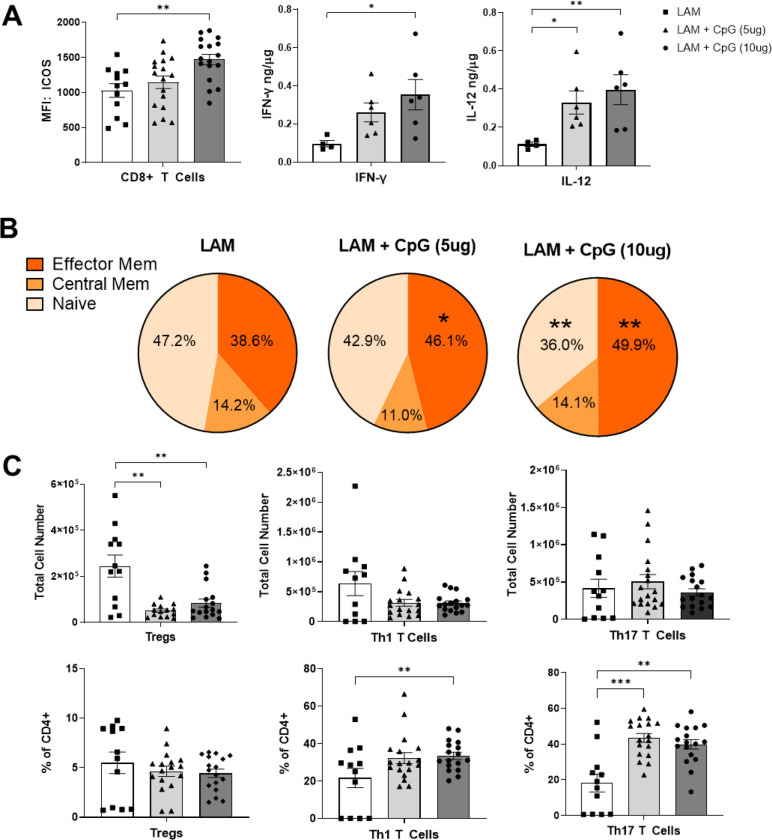
Helper T cells in CpG treated LAM lungs shift away from immunosuppressive phenotypes and CD8 T cells express activation markers. Data shows T cell populations in the left lung lobe of 22 days after LAM induction and 18 days after beginning CpG treatment. **A)** ICOS expression CD8+T Cells and cytokine levels in LAM lungs. **B)** Effector memory, central memory, and naïve CD8+ T cell subsets. **C)** CD4 helper T cell subsets: total cell numbers and % of CD4+ cells. Data is representative of n= 2+ experiments and n>5 mice. Statistical analysis was performed using one-way ANOVA. * p<0.05, ** p<0.01, *** p<0.001, **** p<0.0001.

**Figure E2:**
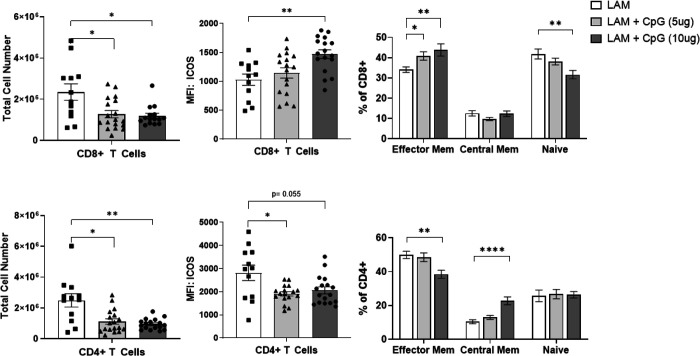
T cells after CpG treatment on day 22. Data shows CD4+ and CD8+ T cell numbers, ICOS expression, and cell population distribution between effector memory, central memory and naïve subsets. Data is representative of n= 3 experiments and n>10 mice. Statistical analysis was performed using one-way ANOVA. * p<0.05, ** p<0.01, *** p<0.001, **** p<0.0001.

**Figure E3:**
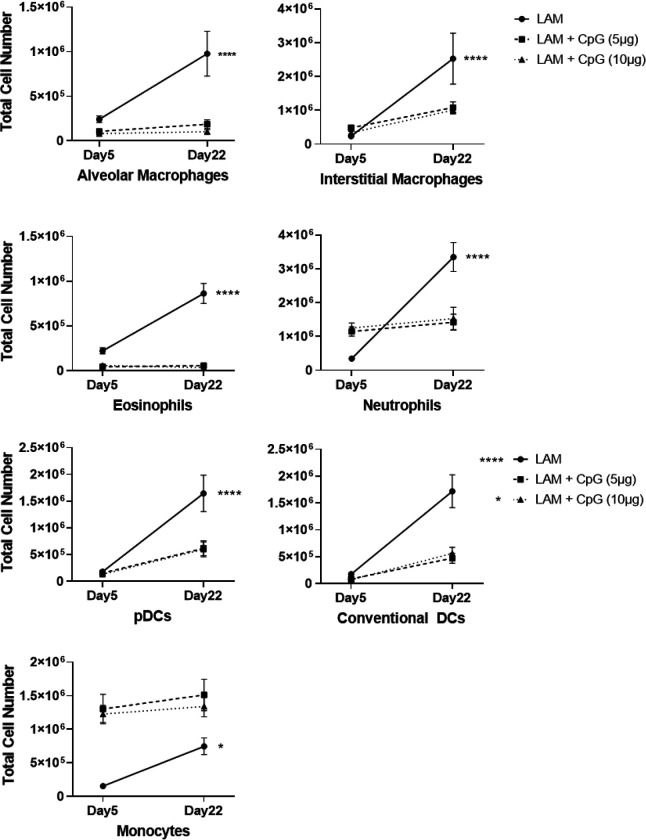
Total cell counts of granulocytes and antigen presenting cells on day 5 and 22 after CpG treatment. Statistical analysis was performed using two-way ANOVA. * p<0.05, ** p<0.01, *** p<0.001, **** p<0.0001.

**Figure E4:**
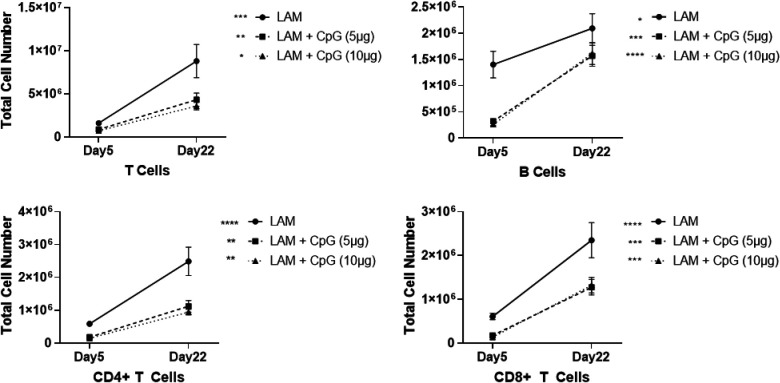
Total cell counts of T and B cells on day 5 and 22 after CpG treatment. Statistical analysis was performed using two-way ANOVA. * p<0.05, ** p<0.01, *** p<0.001, **** p<0.0001.

Although overall CD4+ and CD8+ T cell numbers were not different between treated and untreated LAM lungs (Figure E2), CpG treated LAM lungs show signs of reduced immunosuppression. Flow cytometry analysis reveals that CD8+ T cells have increased expression of activation marker ICOS (Fig 3A) and the proportion of effector memory CD8+ T cells is significantly increased in our CpG treatment groups (Fig 3B). This suggests that CpG treatment activates cytotoxic CD8+ T cells. Cytotoxic T cells are the main producers of IFN-γ, so we measured IFN-γ in lung tissue homogenates by ELISA and found that CpG-treated lungs have increased levels of IFN-γ (Fig 3A), suggesting increased cell-mediated cytotoxicity.

Among CD4+ helper T cells subsets, we found significantly decreased regulatory T cell numbers in CpG treated mice (Fig 3C). We did not see decreases in populations of Th1 or Th17 cells (Fig 3C), therefore we can attribute the overall decrease in CD4 T cells in the CpG treatment groups to a decline in regulatory T cells. This is a positive indicator of decreased immunosuppression. Furthermore, we found increased proportions of Th1 and Th17 cells (Fig 3C) suggesting that T cells in CpG treated LAM lungs are polarized towards more proinflammatory phenotypes.

### Late intervention CpG treatment in LAM mice

**Figure 4.**
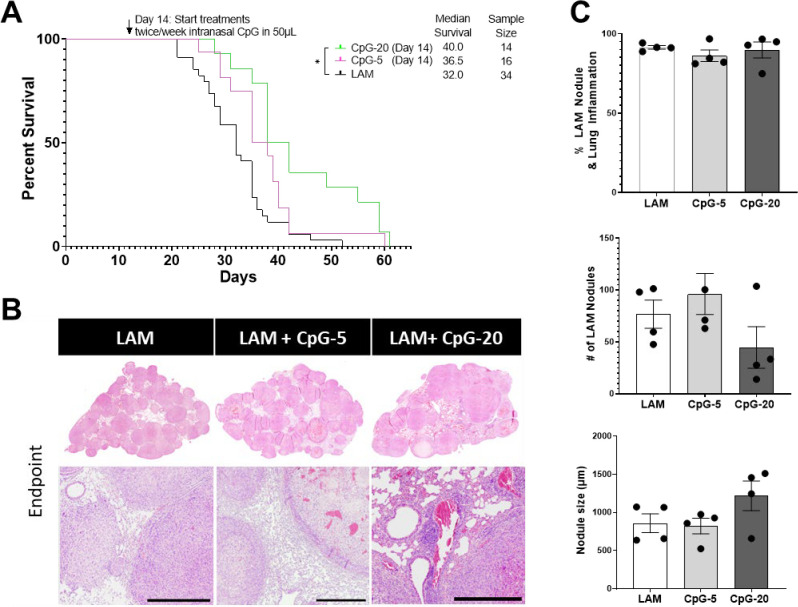
Late intervention intranasal CpG increases survival in murine model of metastatic LAM at higher dose. **A)** Aggregate data from three survival experiments LAM n=34, CpG (10ug ) n=14, CpG (5ug ) n=16. **B)** Representative H&E stained histological images of mouse lungs from treatment groups: untreated LAM control, CpG 5 μg, and CpG 20 μg. Scale bar = 500 μm. **C)** Quantification of LAM nodules and Inflamed tissue regions between treatment groups of mice at symptomatic endpoints from survival studies in (A). 3 sections analyzed per mouse. Statistical analysis was performed using log-rank test (survival studies) and one-way ANOVA (histological analysis). * p<0.05.

Given the encouraging results in administering CpG early in LAM disease, and since LAM can be diagnosed at a variety of disease stages, we next evaluated CpG’s therapeutic potential in more advanced disease. We induced LAM in mice and allowed disease to develop for 14 days, before beginning intranasal treatment with CpG. We found that lower dose CpG (5 μg) had no significant effect on survival (Fig 4A), but a higher dose of CpG (20 μg) showed some therapeutic benefit (Fig 4A). We also found that the CpG treatment did not affect tissue inflammation, nodule count, or nodule size in more advanced disease (Fig 4B). However, we did observe that nodules in CpG-treated mice showed histological signs of necrosis in LAM nodules, unlike untreated LAM controls (Fig 4A)

**Figure 5.**
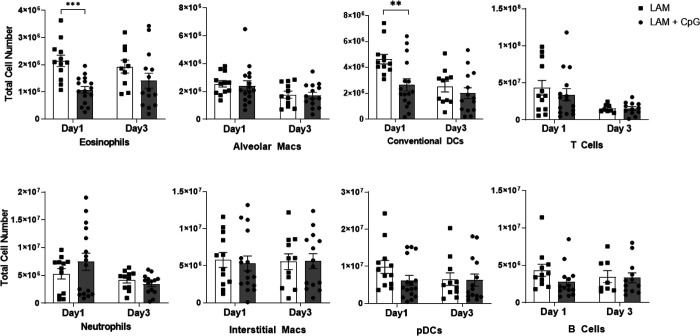
CpG treatment modulates immune cell populations in response to late intervention (starting at day 14) intranasal CpG in LAM lungs (Day 1 & Day 3 after CpG treatment). Total cell numbers of granulocytes and antigen presenting cells infiltrating the lungs 1 and 3 days after CpG treatment obtained via flow cytometry. Data is representative of n= 3 experiments and n = 8+ mice. Statistical analysis was performed using one-way ANOVA. * p<0.05, ** p<0.01, *** p<0.001.

We then compared the immune response in CpG treated LAM mice with advanced disease to untreated LAM controls. We evaluated the effects of CpG treatment on the immune response 1 and 3 days following the first intranasal treatment of CpG given 14 days post disease induction. Unlike the striking responses seen during early intervention, CpG given later in disease did not significantly affect immune cell infiltration in LAM lungs. We found only a decrease in eosinophils and cDCs at day 15; all other populations of identified immune cells remained unchanged (Fig 5).

### Combination CpG and anti-PD1 therapy work synergistically to increase Survival in Murine LAM

**Figure 6.**
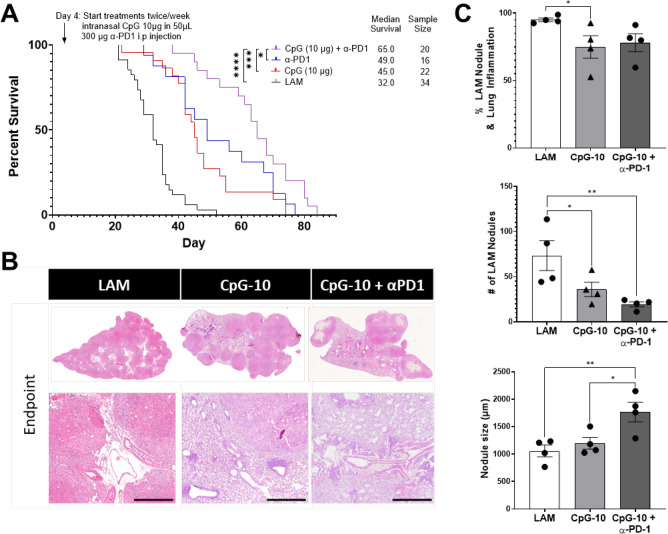
CpG treatment is synergistic with ICI Therapy. **A)** Aggregate data from four survival experiments LAM n=34, CpG (10 μg) n=22, α-PD-1 n=16, CpG (10 μg) + α-PD-1 n=20. **B)** Representative H&E stained histological images of mouse lungs from treatment groups: untreated LAM control, CpG (10 μg), α-PD-1, CpG (10 μg) + α-PD-1 . Scale bar = 500 μm. **C)** Quantification of LAM nodules and Inflamed tissue regions between treatment groups of mice at symptomatic endpoints from survival studies in (A). 3 sections analyzed per mouse. Statistical analysis was performed using log-rank test (survival studies) and one-way ANOVA (histological analysis). * p<0.05, ** p<0.01, *** p<0.001, **** p<0.0001.

We have previously shown that treatment with anti-PD-1 antibody was effective at prolonging survival in murine LAM (17). In addition, CpG has been shown to enhance anti-tumor immunity in pre-clinical cancer models when given in combination with chemotherapy or checkpoint inhibition(32–34). Therefore, we conducted a survival study to evaluate combination therapy of CpG with checkpoint inhibition to determine potential synergistic effects in LAM treatment. We found that combination CpG and anti-PD-1 therapy increased survival in mice (Fig 6A) compared to CpG or anti-PD1 treatment alone. The median survival in mice receiving combination therapy was 65 days compared to 45 days in the CpG treatment group and 49 days in the anti-PD1 treatment group. This suggests that that TLR9 activation can make LAM more responsive to checkpoint inhibition. Histological analysis of tissues collected at symptomatic endpoints showed that combination CpG and anti-PD-1 therapy resulted in reduced LAM nodule counts but had no effect on overall inflammation as a result of increased nodule sizes (Fig 6B–C). We observed signs of necrosis (areas with less nuclear hematoxylin staining) in these larger nodules (Fig 6B ) that were not present in nodules of untreated LAM controls. Tumor necrosis is often associated with enhanced cytotoxic T-cell responses as a result of immune checkpoint inhibition.

## DISCUSSION

Our research offers preclinical evidence to support the benefit of adjuvant-mediated immunotherapy in the treatment of LAM. We found that intranasal treatment with CpG at early stage disease increased survival in mice with LAM. This treatment slowed the progression of disease in the lungs, and decreased the LAM nodule burden in treated mice while increasing the population of cytotoxic and effector CD8+ T cells and decreasing the population of regulatory T cells in the LAM microenvironment. We have previously shown that anti-PD-1 therapy increases survival in mice with LAM and others have found that in cancer immunotherapy, checkpoint inhibitors can synergize with immune adjuvants. Here, we observed synergistic effects of systemic checkpoint inhibition when combined with local CpG adjuvant therapy. This finding suggests that immune activating therapies, particularly combination therapies, could be an effective strategy to treat LAM.

### Striking the balance between good/bad inflammation

A sophisticated balance of pro- and anti-inflammatory signals helps regulate immune responses in the body. Immunotherapies influence these pathways, tipping the scale towards inflammation to produce impressive anti-tumor responses. However, the inflammation induced can also lead to immune mediated adverse effects and inflammatory toxicities. These side effects have hindered the development of immunotherapies as alternative cancer treatments. Choosing the right dose and treatment schedule to maintain equilibrium between treatment efficacy and runaway inflammation could be key to avoiding undesirable tissue damage and severe symptoms. Our finding that lower dose CpG therapy produces less inflammation in the lungs and increases survival in LAM mice despite the increase in nodule count (compared to higher dose CpG), suggests that not all inflammation is helpful for the anti-LAM immune response. In fact, too much inflammation can cause damage in an already delicate tissue environment and be detrimental to treatment outcomes. A recent clinical trial looking at inhaled TLR9 agonist for the treatment of lung cancer reported several adverse events in patients all related to inflammatory immune responses (NCT03326752) (35). For CpG, lower doses may be effective to reign in inflammation while stimulating enough of an inflammatory immune response to drive anti-LAM immunity. Nanoparticle drug delivery techniques can also be employed to target even smaller doses of CpG to specific cell types, which could reduce tissue-wide inflammation while enhancing therapeutic immune responses to inhibit LAM nodule proliferation. Because LAM nodules are composed of many cell types including fibroblasts that may contribute to LAM pathology (36–38), targeting treatment to immune cells could minimize stromal cell exposure to CpG stimulation and curb fibrosis and collagen deposition (tissue remodeling) (39).

The timing of drug administration is another variable in optimizing treatment efficacy. In cancer treatment, immunotherapies like immune checkpoint inhibitors are commonly administered to patients at a high dose every 2–3 weeks, while researchers typically administer these drugs to mice twice weekly. This striking difference suggests that further pharmacokinetic analyses are required prior to translating LAM immunotherapies from mice to humans. In clinical trials assessing safety and efficacy of TLR9 agonists for cancer, the most common dosing schedule was 1–15 mg CpG once per week (NCT00070629, NCT00299728, NCT00292045, NCT00031278), while murine studies including ours can vary from giving a single dose of CpG to up to two doses per week. Our study involved repeated stimulation of TLR9 and it is possible that the frequency of CpG dosing in mice may induce a tolerizing immune response known as TLR or endotoxin tolerance. TLR tolerance is characterized by reduced pro-inflammatory cytokine production and transient unresponsiveness of immune cells upon re-exposure to TLR agonists within 5 days of a prior stimulation (40–45). This could hinder the ability of our therapeutic strategy to maintain an active inflammatory response in the lungs. Tolerance could mean an eventual restoration of the LAM microenvironment to its initial immunosuppressive state, which could explain why CpG, while beneficial, is not curative. Researchers found that delaying a secondary stimulation until after the refractory period (5 days) avoided the induction of TLR tolerance (46). Therefore, adjusting the timing of our treatment regimen in future studies to once weekly or every 2 weeks in mice could prolong the therapeutic effect.

### Our results in context of current LAM therapy and potential for clinical translation

#### Combination therapy with existing LAM treatments:

Because Rapamycin is the best treatment currently available to LAM patients, assessing its compatibility with CpG will be critical for consideration in clinical applications. Rapamycin is an immunosuppressive drug and could affect the immune response induced by CpG treatment. More specifically, the mTOR complex 1 is also involved in TLR9 activation through the myD88 signaling pathway, so inhibition of mTOR by Rapamycin treatment could potentially attenuate the inflammatory response induced by CpG treatment in antigen presenting cells. LAM is treated using low dose Rapamycin and how this low dose will interact with immune activating treatments in LAM is an unstudied area. Given that 40% of LAM patients have partial or no response to treatment with Rapamycin (47), TLR9 activation could offer these individuals a therapeutic strategy with similar benefits to standard of care.

#### Translating cancer immunotherapies to LAM:

Several immunotherapies are currently approved for treatment of lung cancer and may be considered for LAM. These include monoclonal antibodies against EGFR and VEGF/VEGFR pathways and immune checkpoint inhibitors targeting PD-1/PD-L1 or CTLA-4. Clinical trials for adoptive T cell therapy and lung cancer vaccines are ongoing (48) and several immunotherapies are approved for advanced lung cancers (49). Similarly, clinical trials for LAM therapies have explored the use of FDA-approved anti-angiogenic and anti-fibrotic therapies targeting pathways involved in the progression of lung cancer and idiopathic pulmonary fibrosis (Bevacizumab, Nintedanib, Saracatinib), but so far with limited success. Immune checkpoint inhibitors have not yet been tested in LAM patients, but there is promising research in mouse models to support this treatment strategy (17,18). Furthermore, our work showing TLR9 agonist treatments can enhance survival in murine LAM could be translated into the clinic in future, particularly since TLR9 agonists have been approved for several cancers and are explored in clinical trials for lung cancer. Finally, results from lung cancer studies suggest that combination of immune checkpoint inhibitors and TLR9 agonists lead to successful anti-lung cancer immune reactivation. Our data suggests that this strategy could be successful in LAM as well and thus there is potential for designing LAM combination immunotherapies in the future.

### Conclusion

Our work demonstrates that TLR9 agonist CpG can enhance survival in a mouse model of LAM and synergizes with anti-PD1checkpoint inhibitor immunotherapy. Our work highlights a new potential area to develop as LAM treatments, combination immunotherapies, and lays the foundation for clinical translation of LAM vaccines.
